# Different Immune Responses of the Lymphoid Organ in Shrimp at Early Challenge Stage of *Vibrio parahaemolyticus* and WSSV

**DOI:** 10.3390/ani11082160

**Published:** 2021-07-21

**Authors:** Fuxuan Wang, Shihao Li, Fuhua Li

**Affiliations:** 1 Key Laboratory of Experimental Marine Biology, Institute of Oceanology, Chinese Academy of Sciences, Qingdao 266071, China; wangfuxuanzi@163.com (F.W.); fhli@qdio.ac.cn (F.L.); 2 Laboratory for Marine Biology and Biotechnology, Qingdao National Laboratory for Marine Science and Technology, Qingdao 266237, China; 3 Center for Ocean Mega-Science, Chinese Academy of Sciences, Qingdao 266071, China; 4 The Innovation of Seed Design, Chinese Academy of Sciences, Wuhan 430072, China

**Keywords:** lymphoid organ, immune response, *Vibrio parahaemolyticus*, WSSV, *Litopenaeus vannamei*

## Abstract

**Simple Summary:**

Disease is a frequently encountered problem in aquaculture, which always causes global economic losses. White spot syndrome virus (WSSV) and *Vibrio parahaemolyticus* are two of the most destructive pathogens causing severe loss of shrimp aquaculture. Understanding the host immune responses against different pathogens is vital for developing effective disease control technologies. The lymphoid organ is a vital part of the shrimp immune system and exhibits important immune functions including cellular and humoral immunity. However, the immune function of the lymphoid organ and its responses against different pathogens are still largely unclear. In the present study, transcriptomic analysis was applied to compare the differentially expressed genes (DEGs) in the lymphoid organ of shrimp after *Vibrio* or WSSV challenge. Data showed that *Vibrio* challenge induced broad immune responses in the lymphoid organ including activation of several pattern recognition receptors, the proPO activating system, phagocytosis related genes, and immune effectors. In contrast, the immune responses seemed to be inhibited after WSSV infection. The present study suggests that the shrimp lymphoid organ plays different functions in response to the infection of distinct pathogens at early stage, which provides new insights into the immune functions of lymphoid organ in shrimp.

**Abstract:**

The lymphoid organ is an essential part of the immune system involved in cellular and humoral immune responses in shrimp. However, its roles in the immune responses against different pathogens are still largely unclear. In the present study, transcriptomic analysis was applied to compare the differentially expressed genes (DEGs) in the lymphoid organ of shrimp after *Vibrio* or WSSV challenge. In total, 2127 DEGs were screened in the lymphoid organ of shrimp at 6 h post *Vibrio parahaemolyticus* injection, and 1569 DEGs were obtained at the same time after WSSV challenge. KEGG pathway enrichment analysis of these DEGs revealed that two significantly enriched pathways including “neuroactive ligand–receptor interaction” and “protein digestion and absorption” were responsive to both pathogens. In contrast, “lysosome” was the significantly enriched pathway only in *Vibrio* challenge whereas carbohydrate metabolism related pathways were the significantly enriched pathways only in WSSV challenge. Further analysis on immune-related DEGs showed that *Vibrio* challenge induced broad immune responses in the lymphoid organ including activation of several pattern recognition receptors, the proPO activating system, phagocytosis related genes, and immune effectors. In contrast, the immune responses seemed to be inhibited after WSSV infection. The data suggest that the shrimp lymphoid organ plays different functions in response to the infection of distinct pathogens at the early stage, which provides new insights into the immune functions of lymphoid organ in shrimp.

## 1. Introduction

The Pacific whiteleg shrimp *Litopenaeus vannamei* is one of the most important crustaceans in global aquaculture [1]. During the development of the shrimp aquaculture industry, disease is a frequently encountered problem, which always causes global economic losses. Viruses, bacteria, and even parasites are the main pathogens of shrimp in aquaculture [2]. *Vibrio parahaemolyticus* and white spot syndrome virus (WSSV) are two of the most destructive pathogens causing severe loss of shrimp aquaculture [3,4]. Similar to other invertebrates, shrimp usually relies on its innate immune system including humoral and cellular immunity to fight against the invasive pathogens [5]. Circulating hemocytes play a crucial role both in cellular and humoral immune responses [6,7]. Besides the hemocytes, the lymphoid organ is also an important target providing immune defense against invasive pathogens.

The lymphoid organ, also called Oka, was first identified in *Penaeus orientalis* (now called *Fenneropenaeus chinensis*) [8] and then found in other penaeid shrimp including *Penaeus monodon* [9] and *L. vannamei* [10]. Therefore, the lymphoid organ was once thought to be unique to penaeid shrimps [11]. However, recent studies showed that the lymphoid organ also existed in *Macrobrachium rosenbergii* [12] and *Procambarus clarkii* [13]. The normal lymphoid organ in shrimp is composed of two similar lobes situated ventral-anteriorly to the hepatopancreas, and its size is affected by many factors including developmental stage, animal size, species and health status, and connects directly to the heart through the subgastric artery [11]. The organ filtrates and removes foreign materials from the hemolymph [11].

The lymphoid organ is a vital part of the shrimp immune system and exhibits important immune functions. The lymphoid organ is important for phagocytosis [14], which plays a major role both in bacteriostasis [15] and viral degradation [16] and functions in humoral immunity. In *P. clarkii* and *M. rosenbergii*, some pattern recognition receptors and immune signaling pathways were differentially expressed at the late stages of WSSV infection [12,13]. In *F. chinensis*, one anti-lipopolysaccharide factor (ALF) specifically expressed in the lymphoid organ showed strong antibacterial and antiviral activities, which further suggested the importance of the lymphoid organ in shrimp humoral immunity [17]. However, the immune function of the lymphoid organ is still largely unclear. How does the lymphoid organ response to the infection of pathogens? What are the differences of the lymphoid organ responsive to different pathogens? These questions are still less investigated.

To understand the early immune responses of the lymphoid organ of shrimp against different pathogens, cDNA libraries from the lymphoid organ of the whiteleg shrimp *L. vannamei* challenged by *V. *parahaemolyticus** or WSSV were sequenced using the Illumina Hiseq 4000 platform in the present study. A total of 2127 and 1569 DEGs were identified in the lymphoid organ of shrimp at 6 h post *Vibrio* or WSSV injection, respectively. KEGG signal pathway enrichment analysis displayed the similarities and differences between antibacterial and antiviral immune responses in the organ. The data will enrich our understanding of the molecular mechanisms of the lymphoid organ against early infection of different pathogens.

## 2. Materials and Methods

### 2.1. Immune Challenge and Shrimp Tissues Preparation

Healthy shrimp about 9–10 g were obtained from our laboratory (Qingdao, China) and cultured in tanks filled with aerated seawater. Shrimp were fed thrice a day with artificial food pellets and acclimatized for three days before challenge experiments.

*V. parahaemolyticus* was isolated from diseased shrimp. Bacteria were cultured in trypticase soybroth (TSB) media (17.0 g/L tryptone, 3.0 g/L phytone, 5.0 g/L NaCl, 2.5 g/L KH_2_PO_4_, 2.5 g/L glucose) at 30 °C with shaking at 200 rpm. When the cultured bacteria reached the logarithmic growth phase, formaldehyde solution was added to cultures at a final concentration of 0.5% (*vol/vol*) and set for 24 h at 4 °C. Excess formaldehyde was removed by three washes with PBS. The formalinized *V. parahaemolyticus* cell suspension was stored at 4 °C before injection. In addition, live WSSV particles were extracted from pathologically infected shrimp by differential centrifugation and density gradient centrifugation as previously described [18]. These particles were stored at −80 °C before use.

Forty-five shrimps were randomly grouped into three groups including two experimental groups and a control group. For one experimental group, each shrimp was injected with 10^7^ CFU formalinized bacterial cells. For another group, 1000 copies of WSSV particles suspended in 10 μL sterile PBS were injected in the shrimp abdominal segments III and IV. In the control group, equal volume of PBS was injected. At 6 hpi, the lymphoid organ was collected from each shrimp and frozen in liquid nitrogen for RNA extraction.

### 2.2. RNA Extraction and Illumina Sequencing

The total RNA was extracted with RNAiso Plus (TaKaRa, Kyoto, Japan) according to the manufacturer’s instructions. Equal amounts of the total RNA from five lymphoid organs in each group were pooled as a biological replicate. Each group was set with three biological replicates. The RNA samples from the control group were designated as POka-1~3, while the RNA samples from the experimental groups were designated as VOka-1~3 and WOka-1~3 for the *Vibrio* and WSSV challenges, respectively. All nine pools were used for sequencing at Gene Denovo (Guangzhou, China) following the manufacturer’s protocol (Illumina Inc., San Diego, CA, USA). Briefly, the mRNA with poly (A) was enriched from the total RNA using oligo (dT) magnetic beads and fragmented with ultra-sonication. Then, the first strand cDNA was reverse-transcribed using reverse transcriptase and random primers. The second-strand cDNA was synthesized under DNA polymerase I. Subsequently, sequencing adapters were added to short fragments, and the libraries were sent for sequencing using an Illumina HiSeq™ 4000.

### 2.3. De Novo Assembly and Transcriptome Analysis

High-quality clean reads were obtained after raw reads filtration. Next, all clean reads from three sets of samples were assembled into transcripts using Trinity software [19] with default parameters. The redundant transcripts were removed by clustering in TGICL [20] with default parameters. After de novo assembly was completed, the unigenes were aligned against four protein databases including Nr, KEGG, Swiss-Prot, and KOG using BLASTX at the E-value less than 10^−5^. The best alignment results were used to predict the sequence direction and the coding sequence of the unigenes.

### 2.4. Differential Expression Analysis and Functional Characterization

To calculate gene abundances, clean reads from each sample were mapped to the assembled transcripts using the RSEM software [21]. Gene expression levels were calculated with the fragments per kilobase of exon model per million mapped reads (FPKM) value and DEGs were identified using the DESeq R package. Besides, false discovery rate (FDR) was used to correct for E-value. Genes were described as DEGs based on the RPKM value in POka and VOka groups or POka and WOka groups, followed by a multiple hypothesis testing: FDR < 0.05 and the absolute value of log2 fold change (FC) > 1. For functional analysis of DEGs, the DEGs were enriched in GO functions and KEGG pathways by the Blast2GO program [22] and KAAS (KEGG Automatic Annotation Server) [23], respectively. The genes related to the immune function were identified based on the functional gene profiling analysis.

### 2.5. Data Reliability Confirmed by qRT-PCR Assay

Four DEGs including Unigene0055376 (alpha 2 macroglobulin), Unigene0018885 (solute carrier organic anion transporter), Unigene0024101 (neuroparsin 1), and Unigene0028929 (C-type lectin) were randomly selected for real-time qPCR (qRT-PCR) in three replicates from the POka, VOka, and WOka groups, respectively. Briefly, 1 μg of total RNA was reverse-transcribed using the PrimeScript RT Reagent Kit with gDNA Eraser (Takara, Japan) following the manufacturer’s protocol. The qRT-PCR reactions were carried out using SYBR (TOYOBO, Osaka, Japan) with gene-specific primers designed by PRIMER 5.00 (Premier Biosoft, San Francisco, CA, USA). The information of primers used in this study is shown in Table S1. The 18S rRNA gene was used as the internal reference gene and the relative gene expression levels were calculated by the comparative Ct method with the formula 2^-ΔΔCt^. All the expression data were obtained from at least three parallel tests. Then, the qRT-PCR results were compared with the transcriptome data.

## 3. Results and Discussion

### 3.1. The Basic Information of the Transcriptome

The detailed information of the transcriptome sequencing is shown in Table 1. A total of 152,386,028 reads were obtained from the POka group, 156,938,950 reads were obtained from the VOka group, and 143,090,744 reads were obtained from the WOka group, respectively. After data filtration, both the clean reads percentage and Q20 (percentage of bases the quality of which was greater than 20 in clean reads) in each sample were higher than 95%. Using the Trinity program, the transcriptome assembly yielded a total of 59,583 unigenes, with a N50 of 2103 bp.

### 3.2. Identification and Verification of DEGs

**Figure 1:**
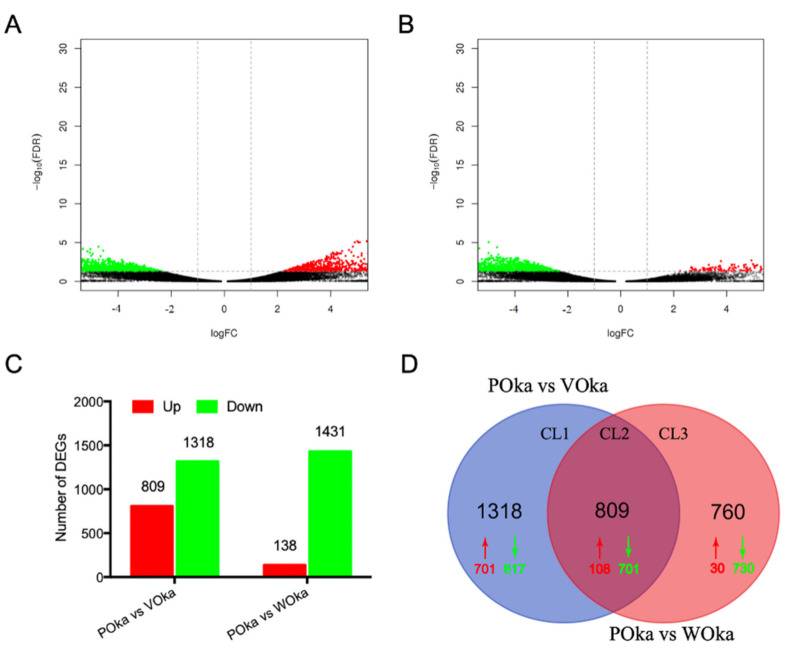
Analysis of the DEGs upon *Vp* and WSSV challenges. (**A**) Volcano plot displaying DEGs between the VOka group and the POka group. (**B**) Volcano plot displaying DEGs between the WOka group and the POka group. The X axis displays the log of the FC (VOka or WOka group relative to POka group) and the Y axis represents the negative log of the FDR (base 10). Black dots represent genes whose expression levels did not reach statistical significance. (**C**) Statistical results of DEGs between the control group and experimental groups. (**D**) A two-set Venn diagram showing the number of DEGs in different treatments. Upregulated genes are marked with red and downregulated genes are shown in green.

To identify DEGs in the lymphoid organ of shrimp after different treatments, the RPKM value was used to compare the expression differences of each gene. According to the definition of DEGs, 2127 DEGs were identified in the VOka group including 809 upregulated DEGs and 1318 downregulated DEGs (Figure 1A,C, Table S2), while 1569 DEGs were identified in the WOka group including 138 upregulated DEGs and 1431 downregulated DEGs (Figure 1B,C, Table S2).

To better understand the immune responses in the lymphoid organ of shrimp caused by *V. parahaemolyticus* and WSSV challenges, a Venn diagram was used to show the DEGs between the VOka group and WOka group. A total of 1318 DEGs in cluster 1 (CL1) and 760 DEGs in cluster 3 (CL3) were specific for the *V. parahaemolyticus* and WSSV challenges, respectively (Figure 1D, Table S2), and 809 DEGs in cluster 2 (CL2) were in the overlapping region (Figure 1D, Table S2), which indicated that these genes might be involved in immune responses against different pathogens.

**Figure 2:**
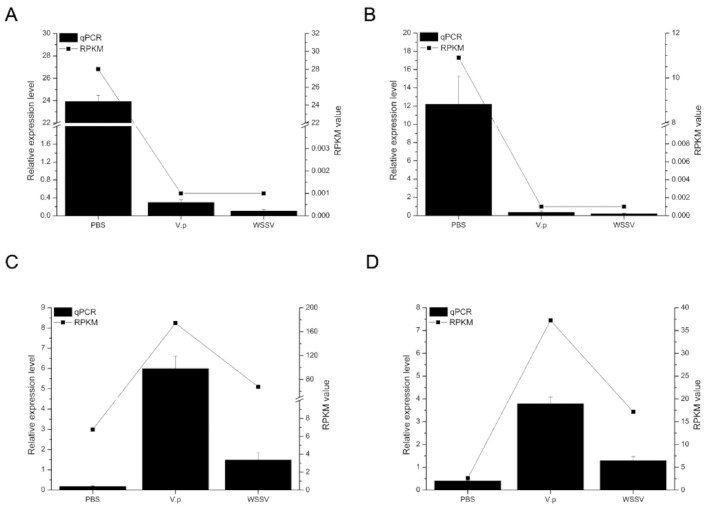
Expression profiles of four unigenes. (**A**) Unigene0055376 (alpha 2 macroglobulin); (**B**) Unigene0018885 (solute carrier organic anion transporter family member 5A1-like); (**C**) Unigene0024101 (neuroparsin 1); (**D**) Unigene0028929 (C-type lectin). The X axis represents the different treatments. Columns and bars represent the means and standard error of relative expression levels from the qPCR results (Y axis at left). Lines represent the RPKM value from the transcriptome results (Y axis at right).

To verify the accuracy of the transcriptome data, four DEGs were randomly selected for qRT-PCR from 809 DEGs located at the intersection of Figure 1D. The expression profiles of them in the transcriptome data were in good agreement with the qPCR results (Figure 2). Unigene0055376 (Figure 2A) and Unigene0018885 (Figure 2B) were significantly downregulated in the lymphoid organ of shrimp under both the *Vp* and WSSV stimulation, while Unigene0024101 (Figure 2C) and Unigene0028929 (Figure 2D) were obviously upregulated.

### 3.3. Functional Characterization of DEGs

**Figure 3:**
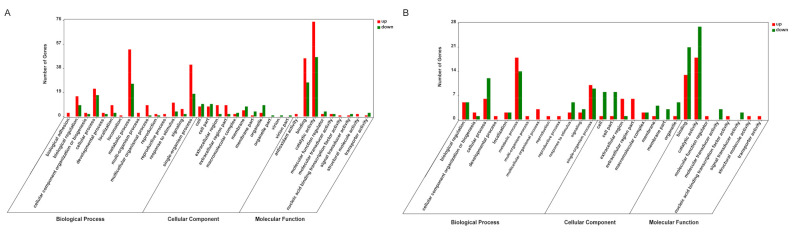
GO term (level 2) distribution for the DEGs from the present transcriptome analysis of lymphoid organ after *V. parahaemolyticus* (**A**) and WSSV (**B**) challenges. The horizontal axis displays the name of GO subcategories. The vertical axis represents the number of genes. Red indicated upregulated DEGs and green showed downregulated DEGs.

To understand the function of DEGs during *V. parahaemolyticus* and WSSV challenges in shrimp, all genes were mapped to terms in the GO and KEGG database. A total of 532 GO terms were successfully enriched in VOka vs. POka (Table S3), and 307 GO terms were enriched in WOka vs. POka (Table S4). The functional distribution of all DEGs is shown in Figure 3. Compared to WOka vs. POka, the level 2 GO terms of VOka vs. POka had some specific terms in each category including biological adhesion in the biological process; antioxidant activity in the molecular function; and virion and virion part in the cellular component (Figure 3). This result provided more information for exploring the differences between bacterial and viral challenges in the lymphoid organ.

**Figure 4:**
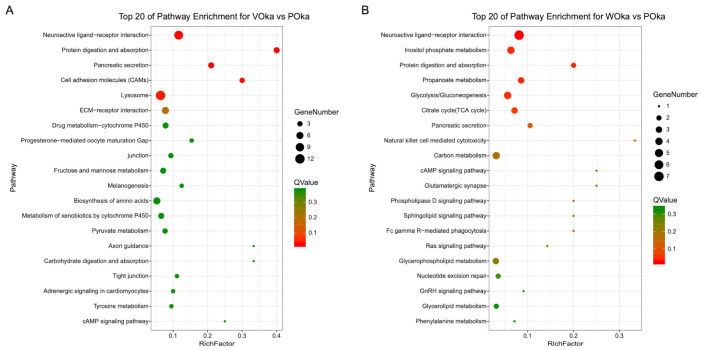
The top 20 KEGG pathways enriched in shrimp lymphoid organ after *V. parahaemolyticus* (**A**) and WSSV (**B**) challenges. Enrichment analysis of KEGG pathways was performed using the KEGG Automatic Annotation Server [23]. “Rich factor” represents the ratio of the DEG number to the number of all genes annotated in this pathway. The Rich factor is proportional to the degree of enrichment.

Signaling pathways were defined using the KEGG database (Tables S5 and S6) and the top 20 pathways were shown in Figure 4. Among them, the top five pathways in the lymphoid organ challenged by two pathogens had significant differences (*p*-value < 0.01 and *q*-value < 0.05) (Figure 4). Importantly, “neuroactive ligand–receptor interaction” and “protein digestion and absorption” were the significantly enriched pathways in both the VOka and WOka groups (Figure 4) and might play important roles in response to pathogen challenge in the lymphoid organ. “Lysosome” was the significantly enriched pathway only in the VOka group (Figure 4, Table S5) and probably served as an important organelle involved in the clearance of invasive *Vibrio*. Carbohydrate metabolism related pathways including “inositol phosphate metabolism”, “propanoate metabolism” and “glycolysis/gluconeogenesis” were the significantly enriched pathways only in the WOka group (Figure 4, Table S6). It had been reported that WSSV could induce changes in carbohydrate metabolism in multiple tissues of shrimp including hemocytes, hepatopancreas, and muscle [24,25]. In shrimp hemocytes, WSSV induces the invertebrate Warburg effect (or aerobic glycolysis) in the early infection stage and significantly activates several metabolic pathways, which is essential for successful viral replication [26]. Our results showed that carbohydrate metabolism in shrimp lymphoid organ was very likely to be altered by WSSV infection.

### 3.4. In-Depth Analysis of Immune-Related DEGs

To further understand the host immune responses in the lymphoid organ against *Vibrio* and WSSV challenges, we identified immune-related DEGs based on their functional annotation. DEGs responsive to *Vibrio* and WSSV challenges were mainly divided into several categories including pattern recognition receptors (PRRs), proPO activating system, signaling pathway, phagocytosis, chitin-binding protein (CBP), antimicrobial peptides (AMPs), etc.

#### 3.4.1. PRRs

In the transcriptome data, 30 transcripts were identified as PRRs. These transcripts were annotated as beta-1,3-glucan-binding protein (BGBP), lipopolysaccharide and beta-1,3-glucan binding protein (LGBP), lectins (mainly C-type lectins, CTLs), NOD-like receptors (NLRs), fibrinogen-related proteins (FREPs), and leucine rich repeat only protein (Table 2). All identified BGBP and LGBP transcripts in DEGs were increased significantly at the early challenge stage of *Vp* while no transcript was changed after WSSV infection (Table 2), indicating the key role of the lymphoid organ in bacteria removal. All CTL transcripts in DEGs were induced obviously by *Vp* challenge, whereas only two of them were responsive to WSSV infection (Table 2). All the other PRRs were only upregulated after *Vp* challenge while no change after WSSV infection, except one transcript encoding FREP2, which was downregulated after WSSV infection while not responsive to *Vp* (Table 2).

#### 3.4.2. ProPO Activating System

In the *Vp*-challenged lymphoid organ, 20 SP transcripts changed significantly, of which 16 were upregulated, whereas in the WSSV-infected lymphoid organs, only eight transcripts changed apparently, of which five were upregulated (Table 3). Like SP, more SPIs were responsive to *Vp* challenge than to WSSV infection (Table 3). Notably, all 12 proPO transcripts including two laccase transcripts and ten hemocyanin transcripts were upregulated by *Vp* challenge, but no transcript changed after WSSV infection (Table 3).

#### 3.4.3. Signaling Pathways

In invertebrates, signaling pathways such as Toll, IMD, JAK/STAT, and RNA interference pathways play important roles in the innate immunity [27,28]. However, only six genes related to immune signaling pathways were differentially expressed after *Vibrio* or WSSV challenge (Table S7). Four of them encoding TRAF6, SOCS2, and AGO4 were responsive to WSSV infection. AGO4 and TRAF6 were downregulated significantly after WSSV infection, whereas SOCS2 was upregulated (Table S7). AGO is an important member of the RNA-induced silencing complex (RISC) in the RNAi pathway [29]. Here, we found that AGO4 was also expressed highly (RPKM = 309.54) in the lymphoid organ in *L. vannamei* and its expression was decreased rapidly to a very low level (RPKM = 0.08) after WSSV infection. TRAF6 is a central signaling adapter protein that activates nuclear factor κB (NF-κB), mitogen-activated protein kinase (MAPK), phosphoinositide 3-kinase (PI3K) and interferon regulatory factor pathways via its TRAF domain and a RING finger domain [30]. The role of TRAF6 in the antiviral response has been shown in two kinds of shrimp [31,32]. SOCS2 is an inhibitor of the JAK/STAT pathway that mediates antiviral immune response, and knockdown of SOCS2 could enhance resistance against pathogens in both *Marsupenaeus japonicus* and *L. vannamei* [33,34]. Therefore, downregulation of AGO4 and TRAF6 and upregulation of SOCS2 in the lymphoid organ might inhibit antiviral function and increase the shrimp body’s susceptibility to WSSV.

#### 3.4.4. Phagocytosis

In the present transcriptome, 14 protease transcripts changed obviously after *Vp* challenge, 12 of which increased, whereas no transcript changed after WSSV infection (Table 4). Seven cathepsin transcripts that belong to three categories (C, D, and L) were significantly induced after *Vp* challenge (Table 4).

#### 3.4.5. Chitin-Binding Proteins (CBPs)

CBPs, which include chitinases, peritrophic matrix proteins, and several other proteins with unknown function, play important roles in immune responses in many peripheral tissues of shrimp such as stomach, epidermis, and gill [35,36,37,38]. In the transcriptome data, CBPs exhibited differential expression under the treatment of two pathogens in the lymphoid organ. Up to 34 transcripts encoding CBPs were responsive to *Vp* or WSSV challenge. Among them, 25 transcripts specifically responded to *Vibrio* challenge, eight transcripts responded to both pathogens, while only one transcript was specific to WSSV challenge (Table S7). Furthermore, all the 14 peritrophin encoding transcripts were upregulated after *Vibrio* challenge and four of them were upregulated after WSSV challenge (Table S7, bolded). However, most of the other CBPs were downregulated after *Vibrio* or WSSV challenge. The data showed that CBPs were also abundantly expressed in the lymphoid organ and played important roles immediately after pathogen infection.

#### 3.4.6. Immune Effectors

Immune effectors are essential factors of the host immune system, which could directly kill invasive pathogens and induce amplified host immune responses. Antimicrobial peptides are one of the important immune effectors in shrimp, which mainly include penaeidins, anti-lipopolysaccharide factors (ALFs), crustins, and stylins [39]. The expression of two ALFs and three crustins was apparently changed after *Vibrio* or WSSV challenge. The two ALFs were only induced by *Vibrio* challenge, while one crustin was induced after challenge with both *Vibrio* and WSSV (Table 5). However, the other two crustins were downregulated after *Vibrio* or WSSV challenge. The data indicated that different antimicrobial peptides in the lymphoid organ were responsive to distinct pathogens in different ways.

It was noted that eight transcripts encoding vitelline membrane outer layer protein I (VMO-I) displayed the same expression pattern that was upregulated in lymphoid organ after *Vibrio* challenge while not responsive to WSSV challenge (Table 5). VMO-I was initially found in the outer layer of the vitelline membrane (VM) from hen yolk [40]. The VM in arthropods also possess a similar structure and function [41,42]. In the lobster, VMO-I from hepatopancreatic tissue was responsible for humoral immune responses against parasitic infection [43]. In the crayfish, VMO-I is a component of the granules in the granular hemocytes [44]. These findings indicated that VMO-I might be a kind of immune effectors in crustacean. Although the mechanism of VMO-I function remains largely unknown, our findings still provide information about the potential function of VMO-I during *Vibrio* challenge in shrimp lymphoid organ.

## 4. Discussion

The present study compared the transcriptome differences of the shrimp lymphoid organ against challenges of different pathogens. We focused on the early immune responses (6 hpi) of the host lymphoid organ against different pathogens, and *in vivo* WSSV propagation needs a much longer time than 6 h post injection [45]. Therefore, we used formalinized bacteria and live viral particles for immune challenges. The data showed that the host early responses, especially the immune responses, differed significantly against *Vibrio* and WSSV challenges. PRRs play a crucial role in the recognition of the pathogen-associated molecular patterns (PAMPs) derived from bacteria, fungi, virus, and parasites, and initiate downstream signaling events. So far, there are 11 types of PRRs reported in shrimp [46]. Some PRRs including BGBP, LGBP, CTLs, and FREPs could be activated by *Vibrio* challenge at the early infection stage. The complex formed by BGBP or LGBP and its corresponding pathogenic ingredient induces degranulation and the activation of prophenoloxidase in shrimp [47]. As a large family of lectins in crustaceans, CTLs show a ubiquitous spectrum of microbe-associated molecular patterns (MAMPs) binding and induce the subsequent immune responses including phagocytosis, activation of prophenoloxidase and respiratory burst [48]. A few shrimp CTLs are involved in the anti-viral response through interaction with viral structural proteins [49,50]. According to their functions, some lectins are regarded as important PRRs and others are thought to act as immune effectors. Activation of these PRRs after *Vibrio* challenge indicated that a rapid immune response occurred during bacterial infection.

Melanization of pathogens is one major and rapid immune response in invertebrates, which depends on the proPO activating system [51]. The recognition of PRRs to PAMPs on the pathogen activates the proPO system [52]. A serine proteinase (SP) cascade is then triggered and converts the inactive proPO into the active PO [52]. SPs constitute one of the families of proteolytic enzymes including clip-domain serine proteinase designated as a proPO-activating enzyme (PPAE) and large numbers of serine proteinase homologues without precise functions in the SP cascade [52]. The active PO produces melanin and toxic reactive intermediates to fight against invasive pathogens [53]. Due to the high toxicity of intermediates in the melanization process, the process is tightly controlled by SPI via inhibiting the SP cascade [52]. Laccase is a member of POs (copper-containing enzymes) and oxidizes *o*-diphenols, *p*-diphenol, and *p*-diamines [54]. In insects, laccase has a variety of biological functions such as degradation of secondary phenolic compounds, metal ion metabolism, cuticle sclerotization, pigment formation, and immune defense [55]. A laccase from *L. vannamei* was reported to be involved in anti-bacterial host defense [56]. Hemocyanin (HMC) acts as an oxygen carrier, a precursor of some AMPs and PO [57]. Until now, HMC-derived PO activity has been characterized in more than 40 species of invertebrates [57]. The data showed that many genes in the proPO activating system including SP, SPI, and proPO were mainly responsive to *Vibrio* challenge. The results were in accordance with the responses of PRRs, which play important roles in triggering the proPO system.

As an evolutionarily conserved process, phagocytosis plays a vital role in both host defense and tissue homeostasis in a wide diversity of organisms. Phagocytosis is a very complex process including particle recognition and internalization, phagosome maturation, and particle destruction [58]. The phagosome matures via fusion with a lysosome that is known as an “enzyme warehouse” and a cellular “digestive system” due to many intrinsic hydrolytic enzymes [59]. Among them, cathepsins are proteases present in all animals as well as other organisms and they could be divided into three subgroups based on the active amino acid residue: cysteine proteases (including types B, C, F, H, K, L, O, S, V, W, and X), aspartic proteases (including types D and E), and serine proteases (including types A and G) [60,61]. Cathepsin C, D, and L were reported to play important roles during immune response against pathogen infection in shrimp or crayfish [62,63,64,65,66]. The present data showed that the lysosome mediated digestion of invasive pathogens was immediately activated in the lymphoid organ during *Vibrio* challenge, whereas this system was not responsive to viral challenge.

## 5. Conclusions

This is the first report on the comparative transcriptome analysis of the shrimp lymphoid organ in response to the early challenge of two different pathogens, *V. parahaemolyticus* and WSSV. *Vibrio* challenge activated broad immune responses including the humoral and cellular immune responses in the lymphoid organ. However, the immune responses seemed to be inhibited in the lymphoid organ at the early infection stage of WSSV. Instead, the carbohydrate metabolism related pathways were induced after WSSV infection. The data suggest that the shrimp lymphoid organ plays different functions in response to the early infection of distinct pathogens, which provides new insights into the immune functions of the lymphoid organ in shrimp.

## Figures and Tables

**Table 1:** Summary of the transcriptome data.

Samples	Raw Reads	Adapter (%)	Low Quality (%)	Clean Reads (%)	GC	Clean Bases (bp)	Q20 (%)
POka-1	50,743,734	39,416 (0.08%)	1,532,076 (3.02%)	49,170,842 (96.90%)	45.41%	7,351,935,942	7,049,298,471 (95.88%)
POka-2	45,500,944	32,062 (0.07%)	1,515,748 (3.33%)	43,952,044 (96.60%)	45.24%	6,571,862,271	6,288,283,729 (95.68%)
POka-3	56,141,350	44,672 (0.08%)	1,919,290 (3.42%)	54,175,832 (96.50%)	46.58%	8,097,101,318	7,747,188,736 (95.68%)
VOka-1	45,722,036	64,476 (0.14%)	1,640,504 (3.59%)	44,016,176 (96.27%)	48.29%	6,573,342,054	6,284,318,401 (95.60%)
VOka-2	61,168,912	81,778 (0.13%)	2,388,808 (3.91%)	58,697,110 (95.96%)	49.48%	8,769,829,446	8,372,020,539 (95.46%)
VOka-3	50,048,002	77,522 (0.15%)	1,890,092 (3.78%)	48,079,500 (96.07%)	49.02%	7,180,638,897	6,859,211,988 (95.52%)
WOka-1	59,908,592	95,602 (0.16%)	2,260,780 (3.77%)	57,551,410 (96.07%)	50.18%	8,599,297,116	8,212,992,611 (95.51%)
WOka-2	41,272,526	55,908 (0.14%)	1,723,184 (4.18%)	39,492,746 (95.69%)	48.27%	5,896,503,470	5,621,053,593 (95.33%)
WOka-3	41,909,626	76,846 (0.18%)	1,458,246 (3.48%)	40,373,928 (96.34%)	50.29%	6,028,800,808	5,764,943,773 (95.62%)

**Table 2:** Differentially expressed PRRs in the lymphoid organ after *Vibrio* or WSSV challenge.

Classification	Gene ID	Nr-Annotation	log2 Ratio (VOka/POka)	log2 Ratio (WOka/POka)
BGBP	Unigene0006001	beta-1,3-glucan-binding protein precursor	9.2689	not significant
	Unigene0006002	beta-1,3-glucan-binding protein precursor	7.2856	not significant
	Unigene0011623	beta-1,3-glucan-binding protein precursor	5.8020	not significant
	Unigene0006003	beta-1,3-glucan binding protein	7.1715	not significant
	Unigene0022216	beta-1,3-glucan binding protein	13.0141	not significant
	Unigene0020907	Beta-1,3-glucan-binding protein	9.9063	not significant
LGBP	Unigene0011641	lipopolysaccharide and beta-1,3-glucan binding protein	5.6731	not significant
CTL	Unigene0021953	antiviral protein	7.1551	not significant
	Unigene0007029	mannose-binding protein	4.1113	not significant
	Unigene0011922	lectin D, partial	7.0167	not significant
	Unigene0001862	lectin B isoform 3, partial	7.1265	not significant
	Unigene0009722	lectin B isoform 3, partial	7.4837	not significant
	Unigene0007283	C-type lectin 5	7.8437	not significant
	Unigene0025672	C-type lectin 4	6.1137	not significant
	Unigene0026420	C-type lectin 2	4.8577	not significant
	Unigene0005566	C-type lectin 1	8.0877	not significant
	Unigene0036730	C-type lectin 1	6.4531	not significant
	Unigene0056556	C-type lectin 1	8.6933	not significant
	Unigene0008649	C-type lectin	3.8902	not significant
	Unigene0042720	C-type lectin	6.5687	not significant
	Unigene0004121	C-type lectin	11.8192	not significant
	Unigene0040089	C-type lectin	5.4285	not significant
	Unigene0042727	C-type lectin	7.9989	not significant
	Unigene0028929	C-type lectin	3.8404	2.7238
	Unigene0029885	C-type lectin	8.7081	4.7517
others	Unigene0045581	Protein NLRC5	2.6992	not significant
	Unigene0037611	protein NLRC5-like	3.1469	not significant
	Unigene0012727	fibrinogen-like protein	4.5223	not significant
	Unigene0022681	FREP2	not significant	−3.8725
	Unigene0038302	leucine rich repeat only protein	7.9370	not significant

**Table 3:** DEGs related to the proPO system in the lymphoid organ after *Vibrio* or WSSV challenge.

Classification	Gene ID	Nr-Annotation	log2 Ratio (VOka/POka)	log2 Ratio (WOka/POka)
SP	Unigene0006596	preprochymotrypsin 1	7.5397	not significant
	Unigene0008624	Chymotrypsin BII	6.7811	not significant
	Unigene0040871	Chymotrypsin BII	6.7003	not significant
	Unigene0032512	chymotrypsin-2-like	6.4923	not significant
	Unigene0032601	trypsin	6.1049	not significant
	Unigene0006379	trypsin	6.3933	not significant
	Unigene0010203	trypsin-1	−5.3595	not significant
	Unigene0007993	trypsin-1	−4.9586	not significant
	Unigene0031678	venom protease-like	4.0204	not significant
	Unigene0007586	CUB-serine protease	4.0236	not significant
	Unigene0024520	clip domain serine proteinase 2	2.8457	not significant
	Unigene0033178	serine protease	3.8166	not significant
	Unigene0009569	transmembrane protease serine 12-like	4.9554	not significant
	Unigene0013356	coagulation factor IX-like	−3.2687	not significant
	Unigene0008625	preprochymotrypsin 1	7.3505	4.2663
	Unigene0040873	preprochymotrypsin 1	6.9669	3.8781
	Unigene0023371	Trypsin-7	−5.6098	−5.9965
	Unigene0006380	trypsin, partial	6.6686	4.7227
	Unigene0042231	trypsin, partial	6.4265	4.6668
	Unigene0006516	trypsin	8.8686	8.7520
	Unigene0007302	serine protease SP24D	not significant	−14.7897
	Unigene0028519	clip domain serine proteinase 1	not significant	−3.5529
SPI	Unigene0004226	inter-alpha-trypsin inhibitor heavy chain H3	7.8262	not significant
	Unigene0037147	Kazal-type serine proteinase inhibitor 4	7.4926	not significant
	Unigene0030648	Kazal-type protease inhibitor	5.3919	not significant
	Unigene0012105	serine proteinase inhibitor-3	−6.3212	not significant
	Unigene0040667	serine proteinase inhibitor 8	3.5000	not significant
	Unigene0040665	serine proteinase inhibitor	−3.7258	not significant
	Unigene0031232	Kunitz-type serine protease inhibitor	4.1047	3.1427
	Unigene0033607	kazal type protease inhibitor	3.1518	2.6125
	Unigene0040669	serine proteinase inhibitor 8	3.8173	3.3256
	Unigene0006314	serine proteinase inhibitor	3.8517	3.4715
	Unigene0028753	serine proteinase inhibitor	4.8338	5.5177
	Unigene0040666	serine proteinase inhibitor	4.1175	3.7476
proPO	Unigene0006825	laccase-1-like isoform X1	5.3797	not significant
	Unigene0040447	Laccase 2	8.2782	not significant
	Unigene0043665	hemocyanin, partial	8.9674	not significant
	Unigene0006113	hemocyanin subunit L4, partial	7.4781	not significant
	Unigene0043668	hemocyanin subunit L4, partial	6.2013	not significant
	Unigene0008173	hemocyanin subunit L3, partial	14.5992	not significant
	Unigene0006336	hemocyanin subunit L2, partial	8.0490	not significant
	Unigene0043663	hemocyanin subunit L2, partial	10.1345	not significant
	Unigene0006337	hemocyanin subunit L1, partial	6.3323	not significant
	Unigene0043667	hemocyanin subunit L1, partial	8.4202	not significant
	Unigene0025329	hemocyanin	6.0904	not significant
	Unigene0043666	hemocyanin	12.5672	not significant

**Table 4:** DEGs related to lysosome in the lymphoid organ after *Vibrio* or WSSV challenge.

Gene ID	Nr-Annotation	log2 Ratio (VOka/POka)	log2 Ratio (WOka/POka)
Unigene0014407	invertebrate-type lysozyme protein	−5.7228	not significant
Unigene0015966	invertebrate-type lysozyme 2	−6.8231	not significant
Unigene0043096	beta-hexosaminidase subunit alpha-like	5.1646	not significant
Unigene0039600	cathepsin L, partial	7.9327	not significant
Unigene0004352	cathepsin L	8.8075	not significant
Unigene0004353	cathepsin L	7.1853	not significant
Unigene0039602	cathepsin L, partial	8.0292	not significant
Unigene0044696	cathepsin C	7.4405	not significant
Unigene0044700	cathepsin C	6.2937	not significant
Unigene0038874	cathepsin D	5.8533	not significant
Unigene0043169	tick legumain	7.3633	not significant
Unigene0043171	tick legumain	8.0169	not significant
Unigene0019904	destabilase I	7.2670	not significant
Unigene0008646	gamma-interferon induced thiol reductase GILT3	5.1848	not significant

**Table 5:** DEGs related to immune effectors in the lymphoid organ after *Vibrio* or WSSV challenge.

Classification	Gene ID	Nr-Annotation	log2 Ratio (VOka/POka)	log2 Ratio (WOka/POka)
ALF	Unigene0031278	anti-lipopolysaccharide factor isoform 6	2.5922	not significant
	Unigene0028455	anti-lipopolysaccharide factor isoform 5	2.5390	not significant
Crustin	Unigene0013411	crustin 3	9.6385	9.3923
	Unigene0053122	crustin Pm5	−10.2677	not significant
	Unigene0023765	CruI-1	not significant	−9.2311
VMOI	Unigene0004307	vitelline membrane outer layer protein I like protein	9.2916	not significant
	Unigene0035626	Vitelline membrane outer layer protein 1	7.9527	not significant
	Unigene0004073	vitelline membrane outer layer 1-like protein	7.0706	not significant
	Unigene0009081	vitelline membrane outer layer 1-like protein	7.8432	not significant
	Unigene0021817	vitelline membrane outer layer 1-like protein	7.9325	not significant
	Unigene0021818	vitelline membrane outer layer 1-like protein	8.1444	not significant
	Unigene0037137	vitelline membrane outer layer 1-like protein	8.0456	not significant
	Unigene0057916	vitelline membrane outer layer 1-like protein	7.3888	not significant

## Data Availability

The datasets generated and analyzed during the current study are included in the Supplementary Materials or available in the NCBI Sequence Read Archive (SRA, https://www.ncbi.nlm.nih.gov/bioproject/713898, accessed on 11 March 2021) with the accession number PRJNA713898.

## Supplementary Materials

The following are available online at https://www.mdpi.com/article/10.3390/ani11082160/s1, Table S1: Primers used for qPCR validation, Table S2: Expression and annotation of DEGs identified from VOka group and WOka group, Table S3: GO enrichment analysis of all DEGs from VOka group, Table S4: GO enrichment analysis of all DEGs from WOka group, Table S5: KEGG pathway analysis of all DEGs from VOka group, Table S6: KEGG pathway analysis of all DEGs from WOka group, Table S7: Differentially expressed genes related to signaling pathways, chitin-binding protein, ECM–receptor interaction, neuroactive ligand–receptor interaction, and cellular stress response in Oka after *Vp*.

## Abbreviations

ALFs	Anti-Lipopolysaccharide Factors
CBPs	Chitin-Binding Proteins
DEGs	Differentially Expressed Genes
HMC	Hemocyanin
PRRs	Pattern Recognition Receptors
RPKM	Reads per Kilobase per Million Reads
VMO-I	Vitelline Membrane Outer Layer Protein I
