# Supplementary material for: Different Immune Responses of the Lymphoid Organ in Shrimp at Early Challenge Stage of Vibrio parahaemolyticus and WSSV
Source: Animals (Basel). 2021 Jul 21;11(8):2160. doi: 10.3390/ani11082160 (PMC8388422; doi:10.3390/ani11082160)
Supplement: Supplementary file 1 [file animals-11-02160-s001.zip › Table S1.pdf]

Table S1 Primers used for qPCR validation.

Gene name	Sequence (5' to 3')	Product size (bp)
18S	TATACGCTAGTGGAGCTGGAA	147
	GGGGAGGTAGTGACGAAAAAT	
Unigene0055376	CAGATGCTCCTCTCTCCCGT	200
	GCCACCGTCTCAGTTTCCAA	
Unigene0018885	GCATTCACGCTCTCCTTTAC	120
	CTGTGAGATGAAGAGCAAGAGA	
Unigene0024101	GGCAGCAGGTGGCAAGAAT	152
	TCAACCGAGAACGAGAAGGAA	
Unigene0028929	CTGGTCTCCTTCGGGTGCC	188
	TCTTCATCCGCCTCCTACTCC	
